# Association between Arsenic Exposure and Diabetes: A Meta-Analysis

**DOI:** 10.1155/2015/368087

**Published:** 2015-04-27

**Authors:** Tzu-Ching Sung, Jhih-Wei Huang, How-Ran Guo

**Affiliations:** ^1^Department of Health Care Management, University of Kang Ning, 188 Anjhong Road, Section 5, Tainan 70970, Taiwan; ^2^Center for Occupational and Environmental Health and Preventive Medicine, National Cheng Kung University, 138 Sheng-Li Road, Tainan 70428, Taiwan; ^3^Department of Environmental and Occupational Health, College of Medicine, National Cheng Kung University, 138 Sheng-Li Road, Tainan 70428, Taiwan; ^4^Department of Occupational and Environmental Medicine, National Cheng Kung University Hospital, 138 Sheng-Li Road, Tainan 70428, Taiwan

## Abstract

Studies on the association between arsenic exposure and diabetes mellitus (DM) yielded inconsistent results. Epidemiologic data on the associations between arsenic exposures via inhalation and DM are limited. Therefore, we conducted a meta-analysis to evaluate the risk of DM associated with arsenic exposure. We searched the related literature through a systematic approach and analyzed the data according to the exposure route (inhalation and ingestion). We used random-effect models to estimate the summary relative risks (RRs) for DM associated with arsenic exposure and used *I* ^2^ statistics to assess the heterogeneity of studies. We identified 38 relevant studies, of which the 32 on the ingestion route showed a significant association between arsenic exposure and DM (RR = 1.57; 95% CI 1.27–1.93). Focusing on the 24 studies in which the diagnosis of DM was confirmed using laboratory tests or medical records, we found that the summary RR was 1.71 (95% CI 1.32–2.23), very close to the overall estimates. We concluded that ingested arsenic is associated with the development of DM, but the heterogeneity among the studies may affect the results.

## 1. Introduction

Arsenic is widely distributed in nature environment and mainly transported through water. It can be found in inorganic and organic forms in the environment. Organic arsenic compounds are much less toxic than the inorganic forms, which are the predominant forms in surface and groundwater reservoirs. Arsenic can easily be released from soil into ground water, depending on the temperature, pH, oxidation reduction potential, dissolved oxygen, and conductivity. For the general population, sources of arsenic exposures include air, drinking water, food, and industry (e.g., arsenic dust and fumes) [1].

Arsenic has been recognized as a human carcinogen for over a century [1], and studies have shown that arsenic exposure via air or drinking water may cause cancers of the lung [2, 3], bladder [4–6], skin [7], and kidney [1, 8]. In the past several decades, the associations between arsenic exposure and human cancers have been observed by many researchers in Taiwan [1, 9, 10]. In addition, arsenic exposure was found to be related to other diseases such as vascular [11] and heart diseases [10, 11]. Among them, some epidemiological studies found that arsenic exposure was associated with an increased risk of diabetes mellitus (DM), including those in Taiwan, Mexico, Bangladesh, Chile, Vietnam, Cambodia, Laos, and Thailand [12–15]. Most of the studies were in developing countries where groundwater was the primary source of drinking water, and the confounding effects caused by traditional risk factors such as diet, obesity, and living habits [16] were minimum. Nonetheless, studies on the associations between arsenic exposure and DM had inconsistent results [14, 17–22]. In addition, although some meta-analyses have been conducted on the associations between inorganic arsenic in drinking water and DM [23, 24], they were mostly on exposures through ingestion, and those on the effects of arsenic exposures through inhalation were limited. Therefore, we conducted meta-analyses to evaluate the risks of DM associated with arsenic exposure through different exposure routes.

## 2. Materials and Methods

### 2.1. Definition of Diabetes Mellitus

Most studies adopted the fasting plasma glucose (FPG) method recommended by the American Diabetes Association (ADA) in 2003 for diagnosing DM [25]. ADA proposed that diabetes can be diagnosed with any one of the following three criteria: a FPG of >126 mg/dL (after no caloric intake for at least eight hours), a casual plasma glucose > 200 mg/dL (taken at any time of day without regard to time of the last meal) with classic diabetes symptoms (increased urination, increased thirst, and unexplained weight loss), or an oral glucose tolerance test (OGTT) (75 g dose) of >200 mg/dL for the two-hour sample. However, some studies were based on self-reported diagnosis.

### 2.2. Protocol of Literature Searching for Meta-Analyses

Meta-analysis is a quantitative review technique that may be used to aggregate the results and to explore and quantify the influence of potential moderating variables within a literature comprised of a variety of research items and methodologies [26, 27].

We conducted a literature search for epidemiological studies worldwide published between January 1, 1980, and January 1, 2014. Two researchers (JWH and TCS) independently searched literature by using academic databases including the PubMed, Web of Science, and Google Scholar. We followed the checklist for data reporting of the PRISMA Statement, [28, 29] except for the funding description of item 27. The eligibility criteria comprised (i) population exposed to arsenic, (ii) diabetes mellitus, (iii) outcome of DM related to arsenic described, and (iv) epidemiological studies. There were a total of 375 articles identified initially.

**Figure 1:**
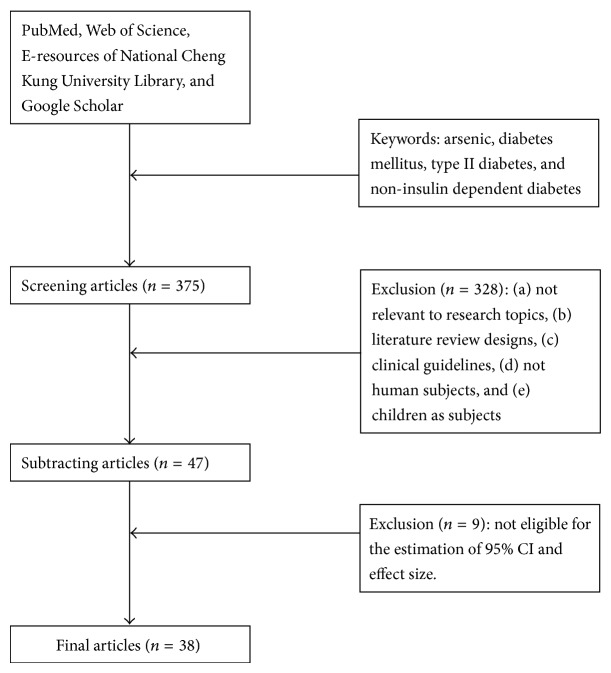
Protocol of references searching.

As recommended by Lipsey and Wilson (2001), the following articles were excluded: (a) not relevant to research topics, (b) literature review designs, (c) clinical guidelines, (d) not human subjects, and (e) children as subjects. Accordingly, 6 review articles (criterion b) and 322 articles (criteria a, c, d, and e) were excluded. In addition, we excluded 9 articles of which the estimates of 95% confidence intervals (CIs) and effect size were not available. As a result, 38 studies were included in our meta-analyses (Figure 1) [13–16, 18, 22, 30–61].

### 2.3. Statistical Analysis

We applied random-effect models (REMs) with the assumption that both within-study and between-study variations exist, which leads to wider and more conservative CIs than fixed effect models (which assume that there is only within-study variation in the mean outcomes of a study and that interstudy variations can be excluded) [62]. Pooled measures were calculated to assess the associations between arsenic exposure and DM, and we conducted separate analyses of studies using arsenic in the drinking water and arsenic in the air for exposure assessment.

Recognizing the fact that the validity of self-reported diagnosis of DM is sometimes questionable, in addition to the analyses that included all relevant studies, we also performed a separate analysis on studies in which the diagnosis of DM was confirmed using laboratory tests, mostly FPG or the oral glucose tolerance test (OGTT), or medical records.

The study-specific adjusted relative risks (RRs) were used as the measure of association across studies. On the basis of the assumption that estimates of odd ratios (ORs) from case-control studies and risk, rate, and hazard ratios from cohort studies were all valid estimates of the RR, we therefore report all results as the RR for simplicity. For those studies that did not use the lowest exposure level as the reference for comparison, we recalculated RRs using the effective count method deriving relative effect and precision estimates for alternative comparisons from a set of estimates presented by exposure level or disease category [63]. To assess the heterogeneity among studies in the metaregression analyses, we calculated the* I* ^2^ statistic [64]. The* I* ^2^ statistic can be interpreted roughly as 0% to 40% indicating that the level of heterogeneity is not remarkable, 30% to 60% indicating moderate heterogeneity, 50% to 90% indicating substantial heterogeneity, and 75% to 100% indicating considerable heterogeneity. All statistical tests were performed at a two-sided significant level of 0.05, and all statistical analyses were carried out using the Comprehensive Meta-Analysis V2 software.

## 3. Results 

There were 38 published studies eligible for meta-analysis on the association between arsenic exposure and DM. We summarized the main characteristics, including route of exposure, case definition, case number, population size, and relative risk with associated 95% CI of each study in Table 1. Most studies were published after 1990, and the earlier studies were mostly on inhalation exposures, 2 out of 3 before 1990, and the latest was back in 2000. The early studies reporting associations between arsenic via drinking water and DM were published in Bangladesh and Taiwan. A relatively large proportion of papers were published after 2010, 10 out of 38 from 2010 to 2014. More than half (*n* = 21) of the 38 studies used the cross-sectional study design, and the rest included 9 cohort studies and 7 case-control studies.

**Figure 2:**
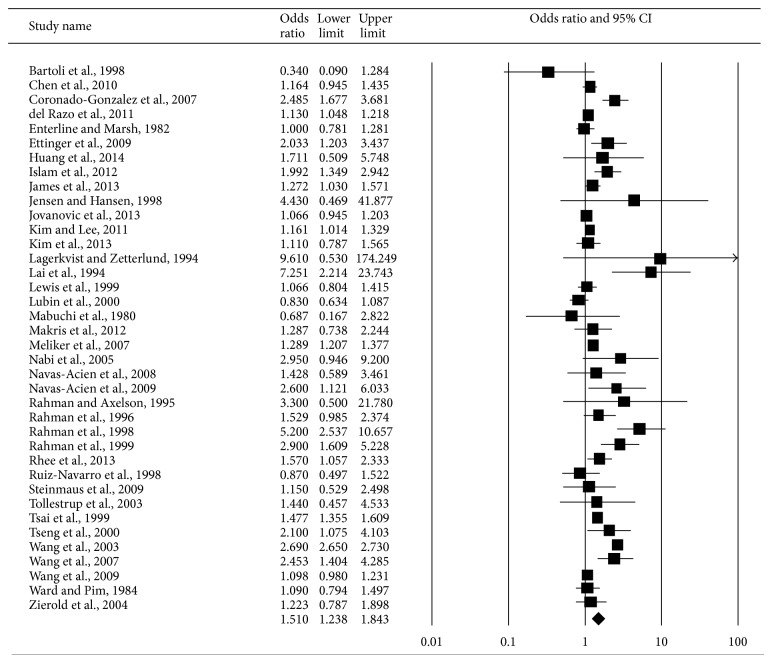
Synthesis forest plot for a random-effect meta-analysis of studies on arsenic exposure and DM. The size of the box is proportional to the weight assigned to each study, which is inversely proportional to the relative risk, and the horizontal line represents the 95% confidence interval.

Of the 38 studies we included in this study, 7 used arsenic in the urine as an indicator of exposure, 18 used arsenic in the drinking water, and 2 used skin symptoms of arsenicosis. Two studies used arsenic in both urine and drinking water as exposure indicators, including one using all three indicators. The summary RR for DM associated with arsenic exposures of these 38 studies was 1.52 (95% CI 1.24–1.85). There was considerable heterogeneity among these studies (*I* ^2^ = 98.17%; *P* < 0.001) (Figure 2).

**Figure 3:**
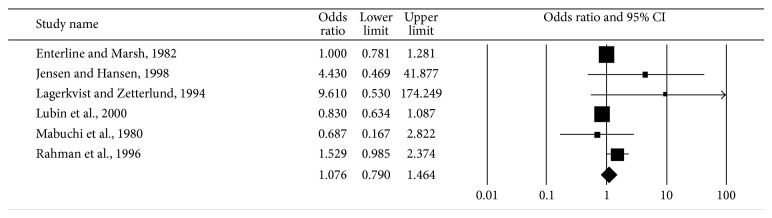
Synthesis forest plot for a random-effect meta-analysis of studies on arsenic exposure through inhalation and DM. The size of the box is proportional to the weight assigned to each study, which is inversely proportional to the relative risk, and the horizontal line represents the 95% confidence interval.

The primary routes of arsenic exposure are ingestion and inhalation, with ingestion as the predominant route (32 out of 38). We conducted a subgroup analysis for these 38 studies stratified into two categories according to the route of exposure: by inhalation and by ingestion. There were six studies [30–35] in the inhalation-route category, and all of them were occupational cohorts: copper smelter workers, wood workers, and pesticide workers. None of the studies had data on exposure level, and the RRs were compared between exposed workers and reference populations, either a group of unexposed workers or the general population. The summary risk did not reach statistical significance (RR = 1.08; 95% CI 0.79–1.46) (Figure 3), and there was moderate heterogeneity among the studies (*I* ^2^ = 48.51%; *P* = 0.084).

**Figure 4:**
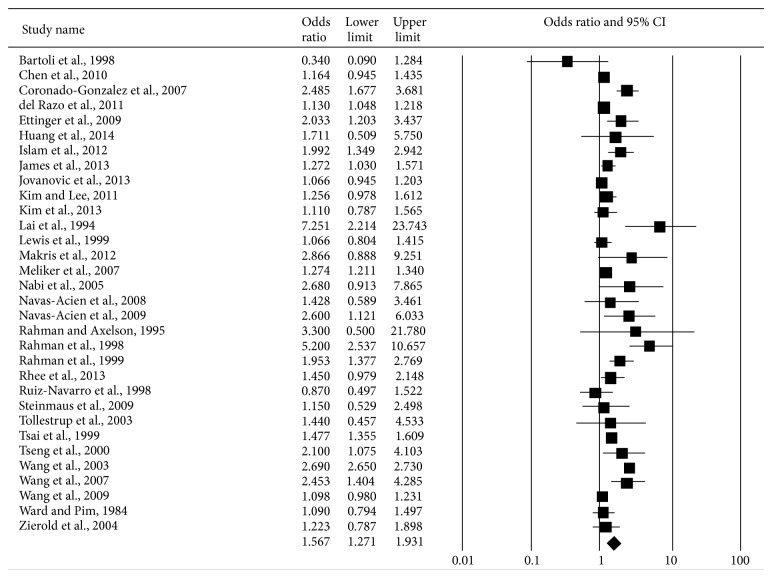
Synthesis forest plot for a random-effect meta-analysis of studies on arsenic exposure through ingestion and DM. The size of the box is proportional to the weight assigned to each study, which is inversely proportional to the relative risk, and the horizontal line represents the 95% confidence interval.

The other 32 studies were included in the ingestion-route category. While some of them simply compared the risks between exposed and unexposed populations, as in the studies on exposures through inhalation, most (28 out of 32) had certain measurements of the exposure levels. Of these studies, the summary RR was 1.57 (95% CI 1.27–1.93) (Figure 4). Likewise, considerable heterogeneity was noted (*I* ^2^ = 98.38%; *P* < 0.001).

**Figure 5:**
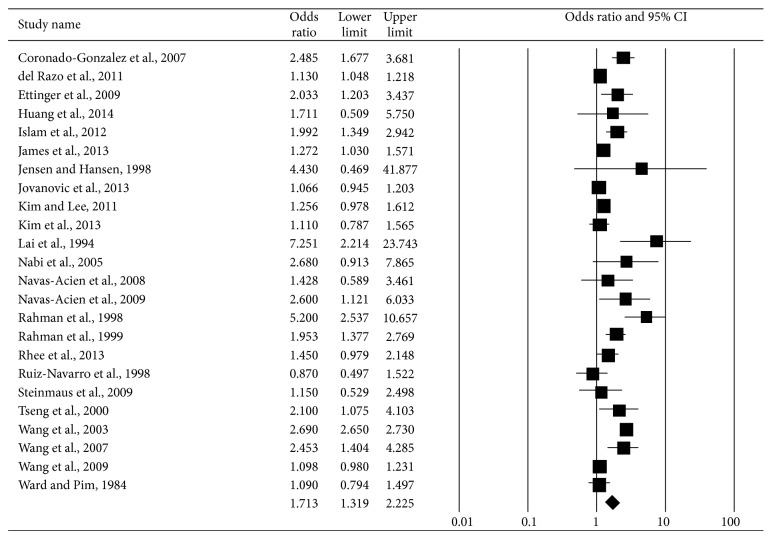
Synthesis forest plot for a random-effect meta-analysis of studies on arsenic exposure through ingestion and DM, in which the diagnosis of DM was confirmed using laboratory tests or medical records. The size of the box is proportional to the weight assigned to each study, which is inversely proportional to the relative risk, and the horizontal line represents the 95% confidence interval.

In 24 studies, the diagnosis of DM was confirmed using laboratory tests or medical records. When we excluded the studies without confirmed diagnoses, the estimated pooled RR was 1.71 (95% CI 1.32–2.23) (Figure 5), similar to that from the overall analysis of studies on arsenic exposures through ingestion. This suggested that the reporting of DM in studies without confirmed diagnoses was accurate in most cases or at least indicated that the misclassifications in the DM status in studies without confirmed diagnoses were nearly random in terms of (independent of) arsenic exposure. However, as in the overall analysis of studies on arsenic exposures through ingestion, considerable heterogeneity was still noted (*I* ^2^ = 97.85%; *P* < 0.001).

## 4. Discussions

Our results support an association between ingested arsenic exposure and DM in humans. No significant associations were observed between arsenic exposure through inhalation and the risk of developing DM. Most studies used the arsenic level in drinking water as an indicator of exposure to assess the association [17, 21, 36, 38, 42, 48, 65–67], and only a recent study used skin changes of arsenicosis as an indicator of exposure to arsenic [16].

The possible mechanisms of inorganic arsenic inducing type 2 DM through interfering with insulin-stimulated signal transduction pathway or with critical steps in glucose metabolism have been investigated by Walton et al. [68]. They recognized that all trivalent arsenicals suppressed expression and possibly phosphorylation of protein kinase B (PKB/Akt). Arsenic trioxide was also found to induce the expression and the phosphorylation of PKB/Akt and inhibit the interaction between PKB/Akt and PPARgamma [69]. PKB/Akt suppresses apoptosis and negatively regulates preadipocyte differentiation. Furthermore, arsenic induced inhibition of adipogenesis may occur in the early stage of terminal adipogenic differentiation which indicated a correlation with C/EBP homologous protein (CHOP10), an endoplasmic reticulum stress response protein [70].

Many studies had a relatively small sample size and might suffer from limited study power, but our meta-analyses obtained a larger statistical power by aggregating studies. To increase the accuracy of the diagnosis of DM, we conducted a separate analysis of studies using laboratory tests or medical records for confirming the diagnosis [25] and found that the summary RR remained about the same.

Due to various reasons such as the fact that studies with small samples sizes or no findings with statistical significance are less likely to be published, meta-analyses may be affected by publication bias. We assessed such bias with visual inspection of the funnel plot, Egger's regression asymmetry test, and Begg's Rank Correlation method by examining the relationship between the standardized treatment effect and the variance of the treatment effect using Kendall's Tau. The results showed indications of publication bias and suggested that caution should be taken when interpreting the results.

The heterogeneities among studies we observed in our analyses might be contributable to a number of characteristics that varied among the studies such as age, gender, study design, design quality, arsenic exposure levels, and covariates. There were distinct differences in the reported effects (statistical heterogeneity), study design (methodological heterogeneity), and characteristics of the participants and outcome measures (clinical heterogeneity). Nonetheless, results of our analyses are consistent with the findings from a previous meta-analysis [24] which showed an increased DM risk associated with arsenic exposure.

Because most previous studies were retrospective or cross-sectional studies, further research efforts should be placed on prospective studies, especially those with better controls of potential confounders. In addition, dose-response assessments should be performed, so that regulation of arsenic levels in drinking water can have a more solid scientific basis. Furthermore, with the existing evidence, we believe that interventional studies should be conducted not only for confirming arsenic as a causal agent of DM but also for preventing DM in the endemic areas of exposure.

## Figures and Tables

**Table 1:** Characteristics of studies.

Study (reference)	Route	Case definition	Exposure indicator	Cases/population
Bartoli et al., 1998 [71]	Ingestion	Death certificate	Living in exposure area	3/488

Chen et al., 2010 [22]	Ingestion	Self-report	Arsenic in drinking water	241/11319

Coronado-González et al., 2007 [13]^∗^	Ingestion	FPG Medical records	Arsenic in urine	200/400

del Razo et al., 2011 [18]^∗^	Ingestion	FPG	Arsenic in drinking water	25/258

Enterline and Marsh, 1982 [72]	Inhalation	Death certificate	Smelter workers versus general population	12/1061

Ettinger et al., 2009 [42]^∗^	Ingestion	OGTT	Arsenic in blood	456

Huang et al., 2014 [16]^∗^	Ingestion	FPG HbA1c	Arsenic in drinking water Arsenic in urine Arsenic exposure skin signs	14/142

Islam et al., 2012 [47]^∗^	Ingestion	FPG Self-report	Arsenic in drinking water	47/1004

James et al., 2013 [40]^∗^	Ingestion	Medical records Self-report	Arsenic in drinking water	141/548

Jensen and Hansen, 1998 [73]^∗^	Inhalation	HbA1c	Exposed versus unexposed workers	64

Jovanovic et al., 2013 [17]^∗^	Ingestion	National registry of diabetes	Arsenic in drinking water	242/195190

Kim and Lee, 2011 [19]^∗^	Ingestion	FPG Self-report	Arsenic in urine Women Men	79/891 77/786

Kim et al., 2013 [57]^∗^	Ingestion	Medical records	Arsenic in drinking water	150/300

Lagerkvist and Zetterlund, 1994 [32]	Inhalation	Self-report	Smelter workers versus unexposed reference	89

Lai et al., 1994 [14]^∗^	Ingestion	Medical history OGTT Self-report	Arsenic in drinking water	86/891

Lewis et al., 1999 [51]	Ingestion	Death certificate	Arsenic in drinking water Women Men	35/961 20/1242

Lubin et al., 2000 [74]	Inhalation	Death certificate	Smelter workers versus US general population	54/5011

Mabuchi et al., 1980 [34]	Inhalation	Death certificate	Pesticide workers versus US general population Men Women	1/197 1/43

Makris et al., 2012 [21]	Ingestion	Self-report	Arsenic in drinking water	317

Meliker et al., 2007 [60]	Ingestion	Death certificate	Arsenic in drinking water Women Men	1612/38722 1249/41282

Nabi et al., 2005 [75]^∗^	Ingestion	Serum glucose	Arsenic in drinking water	24/235

Navas-Acien et al., 2008 [50]^∗^	Ingestion	FPG Self-report	Arsenic in urine	93/788

Navas-Acien et al., 2009 [48]^∗^	Ingestion	FPG Self-report	Arsenic in urine	62/1279

Rahman and Axelson, 1995 [37]	Ingestion	Death certificate	Smelter workers versus unexposed	43/369

Mahfuzar Rahman et al., 1996 [38]	Inhalation	Death certificate	Glassworkers versus unexposed Glassblowers Unspecified glassworkers	6/74 25/135

Rahman et al., 1998 [15]^∗^	Ingestion	OGTT Glucosuria Self-report	Arsenic exposure skin signs	18/1107

Rahman et al., 1999 [76]^∗^	Ingestion	Glucosuria	Arsenic in drinking water	105/1481

Rhee et al., 2013 [39]^∗^	Ingestion	FPG Serum insulin	Arsenic in urine	309/3602

Ruiz-Navarro et al., 1998 [52]^∗^	Ingestion	Medical records	Arsenic in urine	38/126

Steinmaus et al., 2009 [77]^∗^	Ingestion	FPG Self-report	Arsenic in urine	795

Tollestrup et al., 2003 [78]	Ingestion	Death certificate	Living in exposure area Men Women	3/162 1/110

Tsai et al., 1999 [56]	Ingestion	Death certificate	Arsenic in drinking water Men Women	188/11193 343/8874

Tseng et al., 2000 [44]^∗^	Ingestion	FPG OGTT	Arsenic in drinking water	41/446

Wang et al., 2003 [59]^∗^	Ingestion	Medical records	Arsenic in drinking water	5998/706314

Wang et al., 2007 [53]^∗^	Ingestion	FPG	Arsenic in hair	166/660

Wang et al., 2009 [45]^∗^	Ingestion	Medical records	Arsenic in drinking water	235

Ward and Pim, 1984 [79]^∗^	Ingestion	Medical records	Arsenic in plasma	117

Zierold et al., 2004 [46]	Ingestion	Self-report	Arsenic in drinking water	1185

FPG: fasting plasma glucose; OGTT: oral glucose tolerance test; ^∗^the diagnosis of DM is confirmed by laboratory tests or medical records.
